# Ammonium intensifies CAM photosynthesis and counteracts drought effects by increasing malate transport and antioxidant capacity in *Guzmania monostachia*

**DOI:** 10.1093/jxb/ery054

**Published:** 2018-02-15

**Authors:** Paula Natália Pereira, Marília Gaspar, J Andrew C Smith, Helenice Mercier

**Affiliations:** 1 Department of Botany, Institute of Biosciences, University of São Paulo, CEP, São Paulo, SP, Brazil; 2 Department of Plant Physiology and Biochemistry, Institute of Botany, CEP, São Paulo, SP, Brazil; 3 Department of Plant Sciences, University of Oxford, Oxford OX1 3RB, UK

**Keywords:** *ALMT*, ammonium, bromeliad, crassulacean acid metabolism, epiphyte, malate channel, nitrate, nitrogen, tonoplast

## Abstract

*Guzmania monostachia* (Bromeliaceae) is a tropical epiphyte capable of up-regulating crassulacean acid metabolism (CAM) in its photosynthetic tissues in response to changing nutrient and water availability. Previous studies have shown that under drought there is a gradient of increasing CAM expression from the basal (youngest) to the apical (oldest) portion of the leaves, and additionally that nitrogen deficiency can further increase CAM intensity in the leaf apex of this bromeliad. The present study investigated the inter-relationships between nitrogen source (nitrate and/or ammonium) and water deficit in regulating CAM expression in *G. monostachia* leaves. The highest CAM activity was observed under ammonium nutrition in combination with water deficit. This was associated with enhanced activity of the key enzyme phospho*enol*pyruvate carboxylase, elevated rates of ATP- and PPi-dependent proton transport at the vacuolar membrane in the presence of malate, and increased transcript levels of the vacuolar malate channel-encoding gene, *ALMT*. Water deficit was consistently associated with higher levels of total soluble sugars, which were maximal under ammonium nutrition, as were the activities of several antioxidant enzymes (superoxide dismutase, catalase, ascorbate peroxidase, and glutathione reductase). Thus, ammonium nutrition, whilst associated with the highest degree of CAM induction in *G. monostachia*, also mitigates the effects of water deficit by osmotic adjustment and can limit oxidative damage in the leaves of this bromeliad under conditions that may be typical of its epiphytic habitat.

## Teaser

Leaves of *Guzmania monostachia* exposed to ammonium and water deficit showed increased CAM photosynthesis and water deficit tolerance by increasing vacuolar malate channel expression levels, antioxidant enzyme activities, and soluble sugar accumulation.

## Introduction

In plants performing crassulacean acid metabolism (CAM) as a water-saving mode of photosynthesis, atmospheric CO_2_ is taken up at night and fixed by phospho*enol*pyruvate carboxylase (PEPC) into organic acids, mainly malic acid, and accumulated in the vacuole of assimilatory cells (Osmond, 1978; Winter and Smith, 1996; Borland *et al.*, 2009). During the subsequent light period, these organic acids are released from the vacuole and decarboxylated by malic enzyme (ME) or phospho*enol*pyruvate carboxykinase (PEPCK), and the CO_2_ thereby liberated is reduced in the Calvin cycle. CAM has been observed in 33 taxonomic families and is estimated to occur in >6% of all species of vascular plants (Winter and Smith, 1996; Crayn *et al.*, 2015; Silvera and Lasso, 2016). Some plants are able to switch between C_3_ and CAM photosynthesis in response to environmental factors, such as temperature, water, photon flux, and nutrients (Winter *et al.*, 2008, 2015; Freschi *et al.*, 2010; Winter and Holtum, 2011; Pereira *et al.*, 2013). However, little has been discussed about the crosstalk between nutrients and CAM compared with the other environmental factors (Ota, 1988*a*, *b*; Santos and Salema, 1991; Winter and Holtum, 2011).

One of the most recent studies performed on *Calandrinia polyandra* (Montiaceae), a facultative CAM species, revealed that slightly increased nocturnal CO_2_ assimilation could be observed when nitrate fertilization was interrupted for 8 d (Winter and Holtum, 2011). However, Ota (1988a, b) found in *Kalanchoë blossfeldiana* (Crassulaceae) that the highest degree of CAM expression was exhibited in the presence of 1 mM or 5 mM nitrate compared with 1 mM or 5 mM ammonium. Rodrigues *et al.* (2014) demonstrated in the apical portion of leaves of *Guzmania monostachia* (Bromeliaceae), another facultative species, a higher level of CAM expression in the absence of nitrogen compared with the absence of either potassium or phosphorus.

Although previous studies have shown that ammonium provided as the sole nitrogen source to plants can be toxic and can negatively affect photosynthetic rates in some species, such as *K. blossfeldiana* and *Moricandia arvensis* (Brassicaceae) (Winter *et al.*, 1982; Ota, 1988*a*), this phenomenon is species specific and depends on the ammonium concentration supplied (Allen and Smith, 1986; Britto and Kronzucker, 2013). A number of recent studies have revealed examples of higher photosynthetic activity in the presence of ammonium compared with nitrate (Guo *et al.*, 2007; Li *et al.*, 2009; Zhonghua *et al.*, 2011; Hessini *et al.*, 2013). *Spartina alterniflora* (Poaceae), a C_4_ species, was able to maintain maximal photosynthetic rates and stomatal conductance when cultivated on ammonium, possibly due to the increased activity of antioxidant enzymes, which limit oxidative damage, and increased PEPC activity (Hessini *et al.*, 2013). *Oryza sativa* (Poaceae) subjected to polyethylene glycol (PEG) 6000-induced water deficit in a medium containing ammonium showed higher CO_2_ assimilation rates when compared with plants cultivated in the presence of nitrate. In addition, Guo *et al.* (2007) inferred that ammonium nutrition enhances the resistance to water stress in rice. However, little is known about the combined effects of ammonium as a nitrogen source and water deficits in CAM plants.

Central to carbon fixation in CAM plants is the nocturnal accumulation of malic acid in the vacuoles of assimilatory cells. If different nitrogen sources impact on the growth of CAM plants, their effects might therefore be reflected in expression of the key components of the transport system involved in malate transfer across the vacuolar membrane. Acidification of the vacuolar interior is driven by H^+^ transport energized by the tonoplast H^+^-ATPase and H^+^-PPiase, as has been demonstrated for a number of CAM plants including *Kalanchoë daigremontiana* (White and Smith, 1989), *Mesembryanthemum crystallinum* (Barkla *et al.*, 1995), and the bromeliad *Ananas comosus* (McRae *et al.*, 2002). In *Nicotiana tabacum* (Solanaceae), a C_3_ species, Lüttge *et al.* (2000) observed higher relative H^+^ transport rates and vacuolar malate accumulation for plants cultivated in the presence of either 10 mM or 20 mM nitrate compared with 3 mM or 6 mM ammonium; however, relative rates of vacuolar H^+^ transport were found to be unaffected by the nitrogen source for the CAM plant *K*. *daigremontiana*. Transport of malate anions into the vacuole occurs as a charge-balancing process in response to the electrochemical gradient established by the two tonoplast H^+^ pumps, thereby bringing about the net accumulation of malic acid in CAM plants (White and Smith, 1989). Malate transport occurs through an inward-rectifying anion-selective ion channel (Hafke *et al.*, 2003) belonging to the aluminium-activated malate transporter (ALMT) family, which is made up of 14 and 13 genes in *Arabidopsis thaliana* and *Vitis vinifera*, respectively (Kovermann *et al.*, 2007; De Angeli *et al.*, 2013). To our knowledge, however, the relationship between inorganic nitrogen source and expression of the vacuolar transport proteins involved in malic acid accumulation has not yet been investigated in CAM plants.

Here, we show that ammonium is more effective than nitrate in supporting the increased CAM expression observed in the leaves of *G. monostachia* when exposed to drought. Plants supplied with ammonium showed higher transcript levels of the ALMT channel responsible for malate transport into the vacuole, increased accumulation of soluble sugars involved in osmotic adjustment, and higher activities of antioxidant enzymes. These responses may be characteristic of epiphytic bromeliads such as *G. monostachia* that possess water-impounding tanks formed by overlapping bases of the rosulate leaves, and which naturally derive much of their nitrogen as ammonium liberated from decaying organic matter in the tank (Inselsbacher *et al.*, 2007).

## Materials and methods

### Plant material and growth conditions

Plants of *G. monostachia* (L.) Rusby ex Mez var. *monostachia* raised in sterile culture were transferred when ~3 cm tall to pots containing a commercial organic substrate (Tropstrato ‘Vida Verde’) and maintained in a glasshouse in the Department of Botany at the University of São Paulo, Brazil, until reaching the adult phase (~2.5 years). After this period, plants cultivated in pots (13 cm diameter and 7 cm height) were transferred to a controlled-environmental chamber for experiments as described by Pereira *et al.* (2013).

### Water and nutrient deficit treatment

The imposition of water deficit treatment on detached leaves was performed as described by Pereira *et al.* (2013), with modifications. After 30 d of acclimation in the chamber conditions, adult plants that averaged 23.7 ± 0.9 cm tall, with 32.7 ± 4.1 leaves and a tank volume of 40.5 ± 7.3 ml, had their 8th to 12th youngest fully developed leaves excised. Detached leaves were individually transferred to glass flasks so that the cut surface of the leaf blade was immersed in either 10 ml of distilled water (control) or 30% (w/v) PEG 6000 (water deficit), both in nutrient solution containing the macronutrients from Knudson medium (Knudson, 1946) [(NH_4_)_2_SO_4_, Ca(NO_3_)_2_, KH_2_PO_4_, MgSO_4_] and micronutrients from MS medium (Murashige and Skoog, 1962) (MnSO_4_·4H_2_O, ZnSO_4_·7H_2_O, H_3_BO_3_, KI, NaMoO·2H_2_O, CoCl_2_·6H_2_O, CuSO_4_·5H_2_O) and Fe-EDTA (Na_2_EDTA·2H_2_O, FeSO_4_·7H_2_O). The treatments with different nitrogen sources were as follows: without nitrogen source (nitrogen-deficient); 2.5 mM ammonium+2.5 mM nitrate (+NH_4_^+^+NO_3_^–^); 5.0 mM ammonium (+NH_4_^+^), or 5.0 mM nitrate (+NO_3_^–^). The osmotic potential of PEG 6000 solutions, in both the absence and presence of nitrogen sources, was determined using a vapour pressure osmometer (Wescor, USA). The osmotic potential of the media ranged from −0.94 MPa to −1.06 MPa.

Flasks with the detached leaves were maintained in a controlled-environment growth chamber as previously described (Pereira *et al.*, 2013). After 7 d, leaves from all treatments were divided into three portions: (i) basal, corresponding to the part of the leaf that forms the tank in the whole plant and contains lower amounts of chlorophyll; (ii) apical, corresponding to the distal half of the green part of the leaf blade towards the leaf tip; and (iii) middle, corresponding to the more basal half of the green part of the leaf blade. Previous studies have shown that there is a spatial and functional division along the leaf blade of *G. monostachia*. The apex of the leaf is mostly responsible for performing photosynthesis, whereas the basal region is involved in water and nutrient uptake (Freschi *et al.*, 2010; Pereira *et al.*, 2013). The apical portions were used for all biochemical and molecular analyses, since this region of the leaves showed the highest CAM activity under water deficit when compared with the middle and basal portions in previously published studies (Freschi *et al.*, 2010; Pereira *et al.*, 2013). The basal portions of the leaves were used only for the molecular assays, to examine the difference in ALMT transcript expression in the apical and basal portions under different nitrogen treatments. The middle regions were used together with the apical portions for proton transport assays on isolated vacuolar membrane vesicles.

### Measurement of relative water content (RWC)

RWC was determined as previously described (Pereira *et al.*, 2013). Leaf water content was calculated using the formula [(FW−DW)/(TW−DW)]×100% (Martin and Schmitt, 1989), where TW corresponds to turgid weight. Measurements were made in triplicate.

### Organic acid quantification

Organic acid quantifications were made by GC according to Pereira *et al.* (2017*a*). Apical portions of leaf samples (100 mg) were collected 1 h after the start of the light period (dawn) and 1 h before the end of the light period (dusk). Results are expressed as micromole per gram of DW (µmol g^-1^ DW).

### PEPC and MDH activities

PEPC and malate dehydrogenase (MDH) extractions and assays were performed as described by Pereira *et al.* (2013). Apical portions of the leaf samples (1 g) were collected 1 h after the start of the light period (dawn). PEPC and MDH activities were expressed as µmol NADH consumed per minute per gram of DW (µmol NADH min^−1^ g^−1^ DW).

### Soluble sugar quantification

To quantify soluble sugars (glucose, fructose, and sucrose), apical portions of the leaf (samples of 100 mg) were collected 1 h after the start of the light period (dawn) and ground in liquid nitrogen, and subsequently homogenized with 500 µl of MCW solution (methanol, chloroform, and water, 12:5:1, v/v/v) containing phenyl β-d-glucopyranoside as an internal standard (2 mg ml^–1^ methanol), and the samples were then incubated at 60 ºC for 30 min. All samples were centrifuged at 16 000 *g* at 4 ºC for 10 min. The supernatant was collected (50 µl) and dried for 1 h at 60ºC in a CentriVap Vacuum Concentrator^®^ (Labonco, Kansas City, USA). The dried sample was then re-suspended in 25 µl of pyridine and 25 µl of bis(trimethylsilyl)trifluoroacetamide (MTBSTFA), and incubated in a dry bath for 1 h at 75 ºC. A 1 µl aliquot of the incubated sample was used to quantify soluble sugar by GC coupled with a chromatographic system (Shimadzu- QP2010SE, Kyoto, Japan), an Agilent-DB5MS column (30 m, 0.25 mm, 0.5 µm), with helium as a carrier gas in a 1.53 ml min^–1^ flux and an auto sampler (Shimadzu-AOC-20i). The column remained at 100 ºC for 5 min, with a temperature ramp from 100 ºC to 320 ºC at a rate of 8 ºC min^–1^. Injector temperature was 275 ºC, with a total flux of 19.8 ml min^–1^ and linear velocity of 46.0 cm s^–1^. Standard curves for glucose, fructose, and sucrose were used to determine the concentrations of individual soluble sugars in the samples. Results were expressed as micromole per gram of dry weight (µmol g^–1^ DW).

### Antioxidant enzyme activities

Fresh apical portions of the leaf samples (200 mg) harvested at 12 h (middle of the light period) were ground with 2 ml of extraction solution as described by Souza *et al.* (2013). Homogenates were centrifuged at 11 000 *g* at 4 °C for 30 min, and supernatants were kept at −80 °C until analysis. Superoxide dismutase (SOD; EC 1.15.1.1) activity was determined according to Beauchamp and Fridovich (1971) with modifications by Balen *et al.* (2009). Glutathione reductase (GR; EC 1.6.4.2) activity was determined following Shaedle and Bassham (1977). Ascorbate peroxidase (APX; EC1.11.1.11) activity was assayed with the method described by Nakano and Asada (1981), as modified by Weng *et al.* (2007). Catalase (CAT; EC 1.11.1.6) activity was quantified according to Luck (1974) with the following modifications: the reaction solution contained 100 mM potassium phosphate (pH 7.5) and 15 mM H_2_O_2_; the reaction was started by adding 100 µl of leaf extract into the reaction solution; the consumption of H_2_O_2_ was measured at 240 nm in a spectrophotometer at 15 s intervals for 2 min; and CAT activity was calculated with an extinction coefficient of 0.4 mM^–1^ cm^–1^ and expressed as µmol H_2_O_2_ min^–1^ mg^-1^ protein.

### Tonoplast isolation

The method for tonoplast vesicle extraction by differential centrifugation of leaf homogenates was based on Pereira *et al.* (2017*b*).

### Measurement of vesicle acidification

Rates of intravesicular acidification on energization of the tonoplast H^+^-ATPase or H^+^-PPiase were determined according to the quinacrine fluorescence quenching method described by White and Smith (1989) and Pereira *et al.* (2017a, b).

### Protein determination

Protein concentration was measured according to Bradford (1976), using BSA as the standard.

### Primer design and validation

Primers for *ALMT* and reference genes [eukaryotic translation initiation factor (*IF5A2*) and polyubiquitin (*UBQP*)] were designed with Primer 3 Plus software (Rozen and Skaletsky, 2000). All primers were designed based on partial transcript sequences previously obtained from the RNA sequencing of *G. monostachia* (unpublished data). Quantitative real-time PCR (qRT-PCR) amplification efficiencies were calculated with LinReg software. The primer pairs used are shown in Supplementary Table S1 at *JXB* online.

###  RNA extraction, cDNA synthesis, and qRT-PCR

Total RNA was extracted from 100 mg of frozen leaf material (apex and basal portions collected separately at dawn) powdered with liquid nitrogen, with further homogenization using Trizol^®^ Reagent (Invitrogen, Carlsbad, USA) and purification following the clean-up protocol of the PureLink^®^ RNA Mini Kit (Ambion). RNA purity and concentration were determined with a NanoDrop^®^2000c Biophotometer (Thermo Fisher Scientific, Wilmington, USA) before and after the sample clean up. RNA integrity was verified by electrophoresis performed on a 1.0% (w/v) agarose/TBE gel and stained with 0.5 µg ml^–1^ ethidium bromide solution. Genomic DNA contamination was removed from the RNA samples by treatment with DNAse I^®^ (Invitrogen). The cDNA was synthesized using the SuperScript^®^ III One-Step RT-PCR System (Invitrogen) as recommended by the manufacturer. Amplifications were carried out in a total volume of 10 μl with SYBR^®^ Select Master Mix (Applied Biosystems) on StepOnePlus^®^ Real-Time PCR (Applied Biosystems). PCR conditions consisted of an initial heating step at 95 °C for 10 min, followed by 95 °C for 15 s, 40 cycles of 54 °C for 30 s, and 72 °C for 30 s. After cycling, melting curves were run from 60 °C to 95 °C for 20 min, to confirm that a single PCR product was amplified. PCR products were sequenced. The analyses of expression stability of the reference genes were performed with BestKeeper (Pfaffl, 2004). The relative expression level of target genes was calculated as described by Vandesompele *et al.* (2002), with the expression values normalized against the geometric mean of the two reference genes, *IF5A2* and *UBQP* (Pfaffl, 2001). All reactions were performed using three biological replicates and two technical replicates.

### Statistical analysis

All data are presented as mean values ±SD. One-way ANOVA was used to analyse the results. Differences between values under watered and drought conditions were assessed using Student’s *t*-test at *P*<0.05. Significant differences among the different nitrogen treatments were contrasted using the Tukey–Kramer test at *P*<0.05. All measurements were performed using three biological replicates

## Results

### PEG 6000 (30%) induces a water deficit in the apical region of detached leaves

**Fig. 1.**
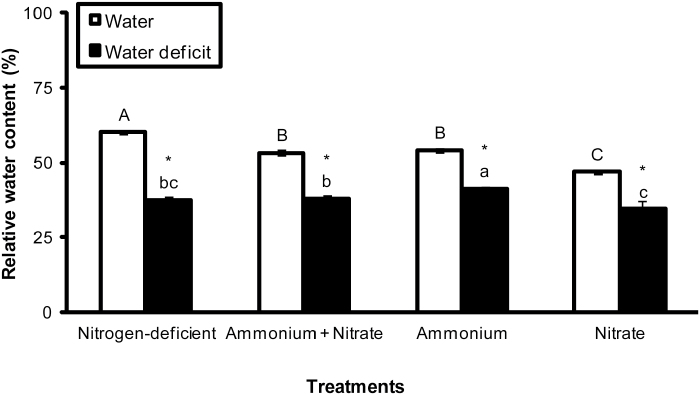
Relative water content in the apical portion of the leaves of *G. monostachia* kept for 7 d in water or water deficit (PEG 6000) associated with the presence or absence of ammonium and/or nitrate. Data are expressed as the mean (±SD) of three replicate samples.

The apical portion of the leaves of *G. monostachia* induced to water deficit for 7 d by the addition of PEG 6000 showed a decrease in the RWC, independent from the nitrogen treatment, when compared with the leaves kept in water (Fig. 1). In addition, NH_4_^+^+water deficit provided a lower decrease in the RWC compared with the other nitrogen treatments under water deficit (Fig. 1).

### Effect of water deficit and nitrogen source on nocturnal organic acid accumulation, and PEPC and MDH activities

**Fig. 2.**
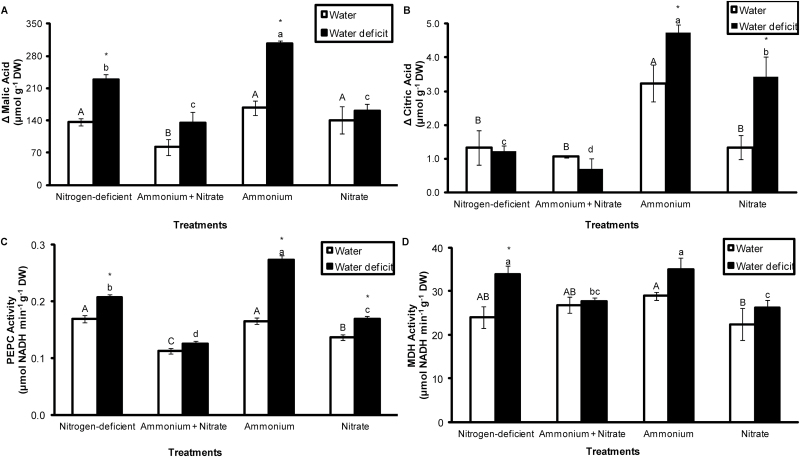
Nocturnal malic (A) and citric (B) acid accumulation, and PEPC (C) and MDH (D) activities in the apical portion of the leaves of *G. monostachia* kept for 7 d in water or water deficit associated with the presence or absence of ammonium and/or nitrate. Data are expressed as the mean (±SD) of three replicate samples

To test the effects of water deficit in combination with different nitrogen sources on CAM photosynthesis in *G. monostachia*, nocturnal organic acid accumulation and the extractable activities of PEPC and MDH were measured in apical portions of leaves. Ammonium nutrition in combination with water deficit elicited the highest degree of nocturnal malate accumulation, followed by the nitrogen deficiency treatment (Fig. 2A). The combination of ammonium and water deficit also produced the highest nocturnal citrate accumulation, but with concentrations of citrate nearly two orders of magnitude lower than for malate (Fig. 2B).

Assays of the extractable activity of PEPC, the key enzyme involved in dark CO_2_ fixation in CAM plants, from the same tissue showed a very similar trend to nocturnal malate accumulation across the different treatments. As expected for this inducible CAM plant, PEPC activity was generally significantly higher in leaves exposed to water deficit compared with the controls; with respect to nitrogen nutrition, the highest PEPC activity was observed in leaves exposed to NH_4_^+^+water deficit, followed by the nitrogen deficiency+water deficit treatment (Fig. 2C). MDH activities, which were approximately two orders of magnitude higher than those of PEPC, did not increase significantly in response to water deficit, except in nitrogen-deficient leaves, whilst in terms of nitrogen nutrition the highest enzymatic activities were again observed in leaves exposed to NH_4_^+^+water deficit and nitrogen deficiency+water deficit conditions (Fig. 2D).

**Fig. 3.**
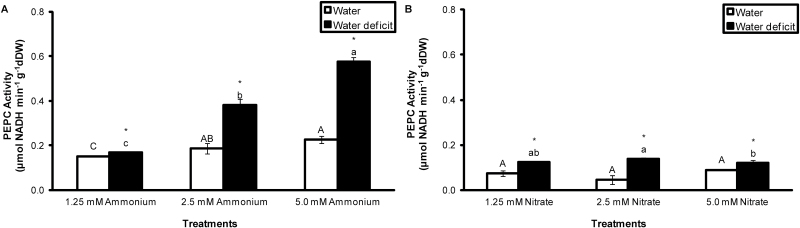
PEPC activity in the apical portion of the leaves of *G. monostachia* kept for 7 d in water or water deficit associated with different ammonium (A) or nitrate (B) concentrations for 7 d. Data are expressed as the mean (±SD) of three replicate samples.

The influence of ammonium and nitrate supplied at different concentrations on extractable PEPC activity was also examined. PEPC activity was always higher in leaves supplied with NH_4_^+^ compared with the equivalent concentration of NO_3_^−^, and PEPC activity increased significantly with increasing ammonium concentration (1.25 mM<2.5 mM<5.0 mM NH_4_^+^: Fig. 3), especially in leaves exposed to water deficit. In contrast, PEPC activity was little affected by differences in nitrate concentration, even under water deficit conditions (Fig. 3B).

### Effect of water deficit and nitrogen source on soluble sugar content

**Fig. 4.**
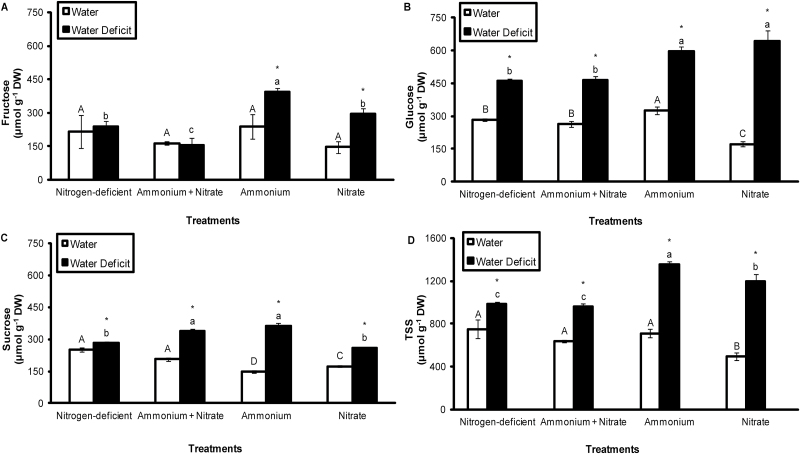
Fructose (A), glucose (B), sucrose (C), and total soluble sugar (TSS) (D) content in the apical portion of the leaves of *G. monostachia* kept for 7 d in water or water deficit associated with the presence or absence of ammonium and/or nitrate. Data are expressed as the mean (±SD) of three replicate samples.

Fructose, glucose, and sucrose were quantified in the apical portions of leaves of *G. monostachia* kept under different nitrogen treatments, and under both control and water deficit conditions, to determine whether these treatments affected the accumulation of other important low molecular weight solutes. Of the soluble sugars, glucose was present at the highest concentrations, followed by fructose and sucrose, depending on the nitrogen treatment and water availability (Fig. 4). In leaf tissue exposed to water deficit, the concentration of soluble sugars, including total soluble sugars (TSSs), was significantly elevated compared with the water controls in almost all nitrogen treatments. With respect to nitrogen treatment, the highest (or joint highest) concentrations of fructose, glucose, and sucrose were observed in leaves supplied with NH_4_^+^ (Fig. 4).

### Effect of water deficit and nitrogen source on activities of antioxidant enzymes

**Fig. 5.**
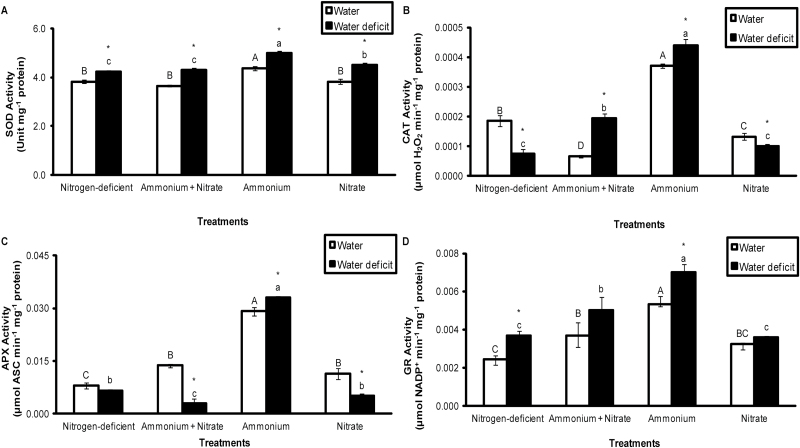
Antioxidant enzymatic activities of SOD (A), CAT (B), APX (C), and GR(D) in the apical portion of the leaves of *G. monostachia* kept for 7 d in water or water deficit associated with the presence or absence of ammonium and/or nitrate. Data are expressed as the mean (±SD) of three replicate samples.

Activities of the four antioxidant enzymes assayed (SOD, CAT, APX, and GR) in the apical portion of leaves of *G. monostachia* were consistently highest in the NH_4_^+^ treatment compared with the other nitrogen sources (Fig. 5). In leaves under water deficit conditions, there was a small but significant increase in SOD activity across all four nitrogen treatments when compared with the water control, whereas for CAT, APX, and GR, whose activities were much lower than that of SOD, a significant increase in enzyme activity in response to water deficit was only consistently observed in the ammonium NH_4_^+^ treatment (Fig. 5).

### Effect of water deficit and nitrogen source on ATP- and PPi-dependent proton transport rates

To investigate the impact of water deficit and nitrogen nutrition on transport activities at the vacuolar membrane associated with CAM expression, vacuolar membranes were isolated by differential centrifugation of leaf homogenates of *G. monostachia*. The relative contribution of vacuolar and non-vacuolar membranes in the membrane fraction isolated from the green portion of the leaf blade (apical+middle portions) was tested using specific inhibitors of the vacuolar H^+^-ATPase (KNO_3_) and non-vacuolar H^+^-ATPases (NaN_3_ and Na_3_VO_4_). This demonstrated that the majority of the ATPase activity measured in the isolated membrane fraction was attributable to the vacuolar membrane (Supplementary Table S2).

To assess the capacity of the vacuolar membrane transporters to drive the accumulation of organic acids, rates of ATP- or PPi-dependent proton transport into isolated membrane vesicles were measured in the presence of fumarate, malate, or citrate as charge-balancing anions for preparations isolated from leaves of plants subjected to the different experimental treatments. Vacuolar membrane vesicles exhibited considerably higher rates of ATP-dependent proton transport in the presence of fumarate compared with malate or citrate (fumarate>malate>citrate) as balancing anions, independent of the nitrogen treatment and water deficit conditions (Table 1). In the presence of fumarate or malate, vesicles from leaves in the NH_4_^+^+water deficit treatment showed the highest rate of ATP-dependent proton transport (Table 1). Across all nitrogen treatments, ATP-dependent proton transport rates in the presence of fumarate and malate were consistently higher in vesicles isolated from leaves exposed to water deficit compared with water controls (Table 1).

**Table 1.** Effect of the charge-balancing anion on initial rates of ATP-dependent proton transport into isolated tonoplast vesicles from leaves (apical+middle portions) of *Guzmania monostachia* kept in water or 30% PEG associated with the presence or absence of ammonium and/or nitrate depending on the balancing carboxylate anion

	Specific activity (% quench min^−1^ mg protein^−1^)		
**Treatments**	**Fumarate**	**Malate**	**Citrate**
Nitrogen deficient+water	162 ± 1.5 Da	72.4 ± 0.3 Cb	35.6 ± 1.3 Cc
Nitrogen deficient+water deficit	197 ± 0.3 Ba	80.3 ± 1.4 Bb	52.7 ± 0.7 Ac
NH_4_^+^+NO_3_^–^+water	164 ± 1.2 Da	59.4 ± 1.0 Db	42.03 ± 0.4 BCc
NH_4_^+^+NO_3_^–^+water deficit	189 ± 6.2 BCa	78.1 ± 1.3 BCb	53.2 ± 1.2 Ac
NH_4_^+^+water	124 ± 1.5 Ea	72.8 ± 3.3 Cb	47.1 ± 1.1 ABc
NH_4_^+^+water deficit	317 ± 1.6 Aa	89.1 ± 0.7 Ab	34.8 ± 1.1 Cc
NO_3_^–^+water	117 ± 3.3 Ea	62.4 ± 1.2 Db	53.4 ± 1.3 Ac
NO_3_^–^+water deficit	182 ± 3.7 Ca	76.4 ± 1.6 BCb	51.2 ± 0.8 Ac

Proton transport was measured as the initial rate of quinacrine fluorescence quenching in the presence of 50 mM fumarate, 50 mM malate, or 50 mM citrate following addition of 3 mM ATP to the suspension of tonoplast vesicles as described in the Materials and methods. Rates of proton transport were quantified as % relative fluorescence quenching min^−1^ mg protein^−1^; values are expressed as means (±SD) for three independent preparations. Different upper case letters indicate values that were significantly different among species using the same anion (Tukey–Kramer test; *P*<0.05). Different lower case letters indicate values that were significantly different among different carboxylate anions in the same species (Tukey–Kramer test; *P*<0.05).

Proton transport into isolated vacuolar membrane vesicles driven by the second H^+^ pump at the vacuolar membrane, the tonoplast H^+^-PPiase, was also dependent on the nature of the carboxylate anion present to provide charge balance, showing the order fumarate>malate>citrate (Table 2). As in the case of ATP-dependent proton transport, the highest rate of proton transport in the presence of fumarate or malate was observed for vesicles from leaves in the NH_4_^+^+water deficit treatment. Moreover, rates of PPi-dependent proton transport tended to be higher in vesicles from leaves exposed to water deficit compared with their respective water controls, and most clearly so in the presence of fumarate as the balancing anion (Table 2).

**Table 2.** Effect of the charge-balancing anion on initial rates of PPi-dependent proton transport into isolated tonoplast vesicles from leaves (apical+middle portions) of *Guzmania monostachia* kept in water or 30% PEG associated with the presence or absence of ammonium and/or nitrate dependinge on the balancing carboxylate anion

	Specific activity (% quench min^−1^ mg protein^−1^)		
**Treatments**	**Fumarate**	**Malate**	**Citrate**
Nitrogen deficient+water	168 ± 5.9 EFa	160 ± 1.2 Ba	144 ± 6.9 Ab
Nitrogen deficient+water deficit	255 ± 7.2 Ba	132 ± 7.7 Cb	122 ± 6.4 Bb
NH_4_^+^+NO_3_^–^+water	107 ± 1.5 Ga	100 ± 5.8 Da	88.9 ± 3.5 Db
NH_4_^+^+NO_3_^–^+water deficit	155 ± 11.1 Fa	117 ± 5.9 Cb	97.9 ± 6.8 CDb
NH_4_^+^+water	216 ± 3.1 Ca	49.9 ± 1.1 Eb	51.01 ± 3.2 Fb
NH_4_^+^+water deficit	293 ± 5.2 Aa	186 ± 5.7 Ab	145 ± 6.8 Ac
NO_3_^–^+water	183 ± 5.4 DEa	185 ± 9.5 Aa	113 ± 4.7 BCb
NO_3_^–^+water deficit	190 ± 5.5 Da	127 ± 1.2 Cb	70.9 ± 6.8 Ec

Proton transport was measured as described in Table 1 in the presence of three different carboxylate anions {fumarate, malate, or citrate, each supplied as their 1,3-bis[tris(hydroxymethyl)methylamino]propane salt at 50 mM}. Rates of proton transport were quantified as % relative fluorescence quenching min^−1^ mg protein^−1^; values are expressed as means (±SD) for three independent preparations. Different upper case letters indicate values that were significantly different among species using the same anion (Tukey–Kramer test; *P*<0.05). Different lower case letters indicate values that were significantly different among different carboxylate anions in the same species (Tukey–Kramer test; *P*<0.05).

### Effect of water deficit and nitrogen source on expression of ALMT

**Fig. 6.**
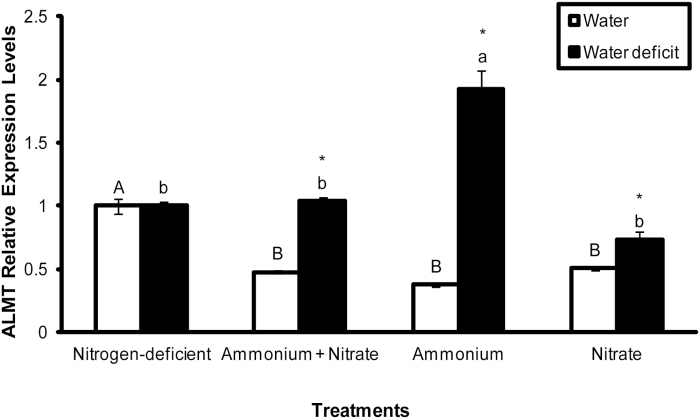
qRT-PCR analysis of the expression of the *GmoALMT* gene in the apical portion of the leaves of *G. monostachia* kept in water or water deficit associated with different nitrogen sources for 7 d. Values represent the expression of *GmoALMT* in plants maintained in water or water deficit and treated with different nitrogen sources relative to their respective control without nitrogen (nitrogen deficient+water or nitrogen deficient+water deficit). The expression levels of *GmoALMT* were normalized using *IF5A2* and *UBQP* as reference genes. Data are expressed as the mean (±SD) of two technical replicates from three biological replicates.

To seek additional evidence of the impact of water deficits and nitrogen nutrition on the vacuolar capacity for CAM-associated malate accumulation, we investigated whether the experimental treatments could be linked to changes at the gene expression level. The most abundant transcript detected in *G. monostachia* leaf tissue from the *ALMT* gene family, encoding anion-selective ion channels known as aluminium-activated malate transporters, was designated *GmALMT1* and its expression was studied by qRT-PCR. Transcript levels of *GmALMT1* were increased by water deficit in all three treatments in which nitrogen was supplied, but most strongly in the NH_4_^+^+water deficit treatment (Fig. 6). This pattern of expression was also found in the basal portion of leaves, where *GmALMT1* transcript was again highest in leaves exposed to NH_4_^+^+water deficit compared with the other nitrogen treatments in combination with water deficit (Supplementary Fig. S1).

## Discussion

The results obtained in this study highlight the important role played by different sources of inorganic nitrogen in controlling the expression of CAM photosynthesis. The epiphytic bromeliad *G. monostachia* has long been known to show drought-inducible CAM activity, especially in the more distal (older) parts of the leaf blade (McWilliams, 1970; Medina *et al.*, 1977; Smith *et al.*, 1985; Maxwell *et al.*, 1992; Freschi *et al.*, 2010; Pereira *et al.*, 2013). The present findings reveal that maximal CAM activity in this species is found when water deficit is combined with ammonium as the inorganic nitrogen source, in comparison with nitrate alone, combined ammonium and nitrate, or nitrogen deficiency. This enhancement of CAM activity under the combined influence of water deficit plus ammonium nutrition was consistently reflected in elevated night-time malic acid accumulation (Fig. 2), PEPC activity (Figs 2, 3), vacuolar transport capacity (Tables 1, 2), and malate channel transcript levels (Fig. 6).

These environmental influences of drought and ammonium on CAM expression are likely to be highly relevant to the fitness and survival of *G. monostachia* in its natural habitat. This species grows as a sun-exposed epiphyte that is subject, at least episodically, to full-intensity sunlight and the effects of an irregular water supply from precipitation (Medina *et al.*, 1977; Smith *et al.*, 1985; Maxwell *et al.*, 1992). Although the water-impounding tanks, or phytotelm, formed by the overlapping leaf bases can buffer the plants against intermittent water supply (Smith, 1989; Benzing, 2000; Males, 2016), the ability to up-regulate expression of the more water-conserving CAM mode of photosynthesis is likely to aid plant survival through periods of intervening water deficits. The further enhancement of CAM activity observed with ammonium nutrition (Figs 2, 3) is also significant in the context of nutrients available to these tank epiphytes in the standing pools of water surrounding the leaf bases in these bromeliads (Benzing, 1970; Benzing and Renfrow, 1974). Specifically, Inselsbacher *et al.* (2007) have shown that ammonium is the most important nitrogen source found in tanks of another water-impounding bromeliad, *Vriesea gigantea*, with a concentration about four times higher than that of nitrate. Ammonium is thus likely to be a major, or even preferred, source of inorganic nitrogen for epiphytic bromeliads with this life form.

Plants vary in their responses to different sources of inorganic nitrogen in a species-specific manner, and this is also true for species performing CAM photosynthesis (see the Introduction). In some there are reports of stimulation of nocturnal CO_2_ fixation by nitrogen deficiency (Ota, 1988*b*; Winter and Holtum, 2011; Rodrigues *et al.*, 2014), and in *K. blossfeldiana* plants it was observed that rates of CO_2_ uptake at night were higher under a low concentration (0.2 mM) of ammonium compared with the same concentration of nitrate (Ota and Yamamoto, 1991). In *G. monostachia*, the highest CAM expression as reflected in nocturnal malate accumulation (Fig. 2A) and PEPC activity (Fig. 2C) was seen at 5.0 mM ammonium compared with 5.0 mM nitrate, and combining ammonium and nitrate in equimolar (2.5 mM) concentrations reduced CAM activity. The decrease in CAM activity in the presence of both nitrogen sources was probably a result of the lower ammonium concentration and the presence of nitrate (Schmitt and Piepenbrock, 1992). Nitrate is known to increase cytokinins levels, which act as a negative regulator of PEPC activity and consequently decrease CAM induction in leaves of *M. crystallinum* (Schmitt and Piepenbrock, 1992) and *G. monostachia* (Pereira *et al.*, 2013). On the other hand, the consequences of ammonium treatment (such as cell acidification) combined with water deficit may contribute to decreasing the levels of cytokinins, increasing ABA content, and increasing PEPC activity, as has been described for other species (Goodchild and Givan, 1990; Pasqualini *et al.*, 2001; Britto and Kronzucker, 2005).

The association between ammonium nutrition and PEPC activity in *G. monostachia* was validated by the positive correlation between ammonium concentration and enzyme activity, which was amplified in combination with water deficit, while PEPC activity was unaffected by nitrate concentration (Fig. 3). A mechanistic explanation for this effect of ammonium concentration may lie in the requirement for enhanced PEPC activity to provide sufficient carbon skeletons via anaplerotic metabolism to support assimilation of ammonium into amino acids (Britto and Kronzucker, 2005, 2013). This elevated activity of PEPC may then permit a higher expression level of CAM when ammonium-supplied plants are subject to water deficit. At first sight, this explanation might appear to conflict with the general observation that malate concentrations tend to be higher in nitrate- compared with ammonium-supplied plants (Lüttge *et al.*, 2000). However, in nitrate-fed plants, malate accumulation is typically charge balanced by a strong inorganic cation such as K or Ca, and can be viewed as a means of transferring excess negative charge resulting from nitrate assimilation to carboxylate as a metabolic end-product. In the case of CAM photosynthesis, however, night-time CO_2_ fixation results in vacuolar accumulation of malic acid, with the malate anions stoichiometrically accompanied by protons. As this process is intrinsically charge balanced, it means that nocturnal malate accumulation in CAM plants is not constrained by the availability of inorganic cations in the same way as applies to carboxylate accumulation in ammonium-fed C_3_ plants.

In addition to elevated PEPC activity and nocturnal malic acid accumulation, the higher CAM activity seen in ammonium-supplied plants of *G. monostachia* exposed to water deficit is also associated with increased capacity for malic acid transport across the vacuolar membrane (Tables 1, 2) and elevated transcript levels for the vacuolar malate channel *GmALMT1* (Fig. 6). This indicates a co-ordinated up-regulation of the enzymatic machinery required for both the nocturnal synthesis and vacuolar accumulation of malic acid under the conditions of nitrogen nutrition and water status conducive to the highest degree of CAM expression. A transport function for a vacuolar-specific isoform of the *ALMT* gene family, *At*ALMT9, was first demonstrated in *Arabidopsis thaliana*, in which it is involved in transport of both malate and fumarate into the vacuole. Transcriptomic studies in CAM plants have also identified highly expressed members of the *ALMT* gene family implicated in CAM-related vacuolar malate transport, for example in photosynthetic versus non-photosynthetic leaf tissue of *Ananas comosus* (Ming *et al.*, 2015) and in the facultative CAM species *Talinum triangulare* exposed to water deficit (Brilhaus *et al.*, 2016). Evidently the full expression of CAM requires not only high activity of the primary H^+^ pumps(s) at the vacuolar membrane (i.e. the H^+^-ATPase and/or H^+^-PPiase; Lüttge *et al.*, 2000), but also the co-ordinated expression of the ion channel responsible for the parallel charge-balancing movement of malate.

Another feature of the leaves of *G. monostachia* supplied with ammonium-nitrogen was evidence of their greater tolerance to drought, as shown by maintenance of a marginally higher RWC compared with other nitrogen treatments under water deficit (Fig. 1), accumulation of the highest total soluble sugar levels (fructose+glucose+sucrose; Fig. 4), and highest activities of antioxidant enzymes (SOD, CAT, APX, and GR; Fig. 5). The accumulation of soluble sugars may not only have osmotic effects in maintaining cell turgor under water-deficit conditions, but may also help to protect membrane integrity and preserve protein functionality (Guo *et al.*, 2007; Krasensky and Jonak, 2012; Lisar *et al.*, 2012; Ceusters *et al.*, 2016). Similarly, the increased activity of antioxidant enzymes may mitigate the effects of oxidative damage that often characterize plant responses to water deficits (Lisar *et al.*, 2012) and help to sustain the higher intensity of CAM in the apical portions of the leaves of *G. monostachia* under these conditions.

In conclusion, this study provides evidence that, in addition to the previously known effect of water deficits, CAM activity in the epiphytic bromeliad *G. monostachia* is also dependent on the nature of the inorganic nitrogen source. The highest CAM expression in this species is observed when ammonium-nitrogen is combined with water deficit, which is associated with a co-ordinated up-regulation of PEPC activity and an enhanced capacity for vacuolar malic acid accumulation. Ammonium also promotes an increase in leaf tolerance to water deficit, probably by osmotic adjustment due to sugar accumulation and by the increased activity of antioxidant enzymes. These metabolic responses appear well suited to the epiphytic habitat of *G. monostachia*, which is characterized by ammonium as the principal source of inorganic nitrogen in the water-impounding tanks, and by an intermittent water supply typical of the sun-exposed niches occupied by this widespread bromeliad.

## Supplementary data

Supplementary data are available at *JXB* online.

**Table S1.** List of oligonucleotides used in this study.

**Table S2.** ATP-dependent proton transport in the presence of an inhibitor of vacuolar or non-vacuolar membranes of *G. monostachia*.

**Fig. S1.** Relative expression of *GmoALMT* in the basal portion of the leaves of *G. monostachia*.

Supplementary Material

## Abbreviations:

ALMT: aluminium-activated malate transporter
APX: ascorbate peroxidase
CAM: Crassulacean acid metabolism
CAT: catalase
GR: glutathione reductase
IF5A2: eukaryotic translation initiation factor
MDH: malate dehydrogenase
PEG: polyethylene glycol
PEPC: phospho*enol*pyruvate carboxylase
RWC: relative water content
SOD: superoxide dismutase
TSS: total soluble sugar
UBQP: polyubiquitin.
