# Supplementary material for: Ammonium intensifies CAM photosynthesis and counteracts drought effects by increasing malate transport and antioxidant capacity in Guzmania monostachia
Source: J Exp Bot. 2018 Feb 15;69(8):1993–2003. doi: 10.1093/jxb/ery054 (PMC6018993; doi:10.1093/jxb/ery054)
Supplement: Supplementary Material [file ery054_suppl_supplementary_material.pdf]

Supplementary Table S1. List of primers used in this study.

Annotation	Primer (Forward/Reverse)	Amplicon length (pb)	Efficiency (%)	R^2
ALMT	5' GCAAAGGATTTAATCGGGGT 3'<br>5' ATAAGGCTTCATCGTCGGGTA 3'	171	91	0.9996
IF5A2	5' TCGGACGAGGAGCACCATTT 3'<br>5' AGTTGAAACTTCCACAACCTTGC 3'	137	90	0.9893
UBQP	5' GATGTTGTAGTCTGCGAGGGT 3'<br>5' TCCAGGACA AGGAGGGGATC 3'	95	84	0.9998

Supplementary Table S2. Effect of ATPase inhibitors on initial rates of ATP-dependent proton transport into isolated tonoplast vesicles from leaves of *Guzmania monostachia* kept in water or PEG 30% associated with presence or absence of ammonium and/or nitrate. Proton transport was measured as described in Table 1 in the presence of 50 mM fumarate following addition of 3 mM ATP to the suspension of tonoplast vesicles. Rates of proton transport were quantified as % relative fluorescence quenching min^{-1} mg $protein^{-1}$. Measurements were made in the presence of inhibitors of vacuolar H^+-ATPase (50 mM KNO_3) or non-vacuolar H^+-ATPases (0.1 mM NaN_3 plus 0.1 mM Na_3VO_4) in comparison with the control (no inhibitors). Results are expressed as means (± s.d.) for three independent preparations. Values in parentheses give the percentage inhibition relative to the control for each species. Different capital letters indicate an average that is significantly different among control, KNO_3 and NaN_3 + Na_4VO_3 in the same treatment ($P < 0.05$; Tukey–Kramer's test). Asterisks indicate significant differences between each inhibitor (KNO_3^- or NaN_3 + $NaVO_3$) and the control within a species by Student's *t*–test at 5% significance level.

	Specific activity (% quench min^{-1} mg $protein^{-1}$) (inhibition relative to control)		
**Treatments**	**Control**	**KNO_3**	**NaN_3 + Na_4VO_3**
Nitrogen-deficient + water	322 ± 18.7 A	122 ± 9.2 C (62%)*	203 ± 17.6 B (37%) *
Nitrogen-deficient + water deficit	365 ±10.2 A	95.8 ± 6.03 C (74%)*	301 ± 10.7 B (17%) *
NH_4^+ + NO_3^- + water	300 ± 8.5 A	33.2 6.2 C (89%)*	188 ± 11.9 B (37%) *
NH_4^+ + NO_3^- + water deficit	297 ± 17.5 A	63.7 ± 4.3 C (79%)*	255 ± 16.8 B (14%)
NH_4^+ + water	263 ± 13.9 A	43.9 ± 9.01 B (83%)*	258 ± 11.5 A (1.9%)
NH_4^+ + water deficit	407 ± 16.9 A	149 ± 14.9 C (63%)*	297 ± 8.9 B (27%) *
NO_3^- + water	117 ± 8.9 A	22.6 ± 2.2 C (81%)*	80.4 ± 4.9 B (31%) *
NO_3^- + water deficit	230 ± 4.9 A	68.4 ± 7.8 C (70%)*	169 ± 9.8 B (26%) *

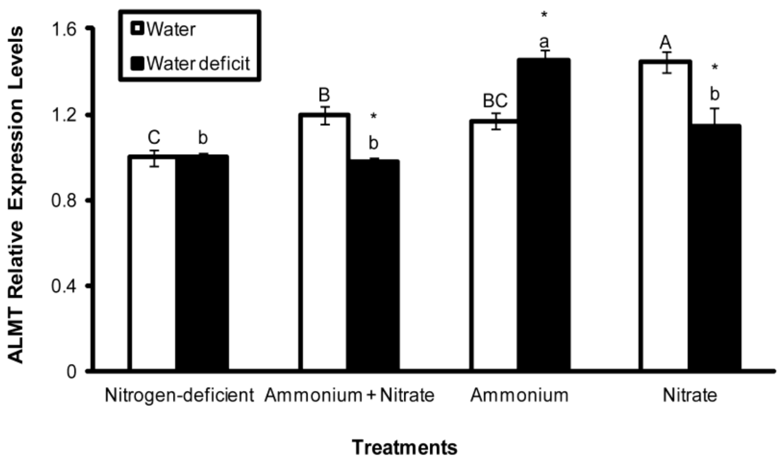

Supplementary Figure S1. RT-qPCR analysis of the expression of *GmoALMT* gene in the basal portion of the leaves of *G. monostachia* kept in water or water deficit associated with different nitrogen sources for 7 days. Values represent the expression of *GmoALMT* in plants maintained in water or water deficit and treated with different nitrogen sources relative to their respective control without nitrogen (nitrogen-deficient + water or nitrogen-deficient + water deficit). The expression levels of *GmoALMT* were normalized using *IF5A2* and *UBQP* as reference genes. Data are expressed as the mean (± s.d.) of two technical replicates from three biological replicates. Asterisks indicate significant differences between water and water deficit in the same nitrogen treatment (*P* < 0.05%; Student's *t*–test). Different capital letters indicate averages that are significantly different among treatments + water in the same portion of the leaves (*P* < 0.05; Tukey–Kramer's test). Different lower case letters indicate averages that are significantly different among treatments + water deficit in the same portion of the leaves (*P* < 0.05; Tukey–Kramer's test).
